# 

**Published:** 1880-05

**Authors:** 


### CONTENTS FOR MAY, 1880.

ORIGINAL COMMUNICATIONS.

PAGE.

ART. I.—Sexual Exhaustion (Neurasthenia). By GEORGE M. BEARD, M. D. .... 221

ART. II.—Electro-Therapeutics. By EDWARD C. MANN, M. D. .... 232

ART. III.—Diagnosis. By B. F. COY, D. D. S. .... 240

ART. IV.—Cold Pack in Scarlatina. By DUDLEY M. CULVER, M. D. .... 242

TRANSLATION.

Dental Caries .... 244

ECLECTIC DEPARTMENT.

Treatment of Subacute and Chronic Gout. By ALEXANDER HADDEN, M. D. .... 247

Dyspepsia and the Teeth .... 253

EDITORIAL DEPARTMENT.

Druggists and Physicians .... 255

Hospitals in Baltimore .... 257

Independent Journalism .... 259

Mortuary Statistics .... 260

"Cat in a Meal Tub." .... 262

Relation of Specialties to each other and to the Profession .... 263

Medical and Chirurgical Faculty of Maryland .... 263

Meetings of Dental Societies .... 264

BIBLIOGRAPHICAL DEPARTMENT .... 264

CORRESPONDENCE .... 268

HYMENEAL. NATALITIAL. NECROLOGICAL. .... 269

Mortuary Statistics and Meteorological Report .... 270

